# Artificial Intelligence-Based Chatbots in Chronic Disease Management: A Systematic Review of Applications and Challenges

**DOI:** 10.7759/cureus.81001

**Published:** 2025-03-22

**Authors:** Dalia Saad Altom, Allaa Ibrahim Awad Taha, Alaa Abu Agla Mahmoud Hussein, Maisoon Ahmed Ibrahim Elshiekh, Ashwag Hassan Alata Abdelmajed, Fatima Ibrahim Abdalla Ibrahim, Sara Mohammed Abelgadir Mohammed, Mosab Mohamed Elamin Eltain Tifoor

**Affiliations:** 1 Family Medicine, Najran Armed Forces Hospital, Ministry of Defense Health Services, Najran, SAU; 2 General Practice, Wad Madani Hospital, Wad Madani, SDN; 3 General Practice, Mediclinic Airport Road Hospital, Abu Dhabi, ARE; 4 Nephrology, Gezira Hospital for Renal Disease and Surgery, Wad Madani, SDN; 5 Internal Medicine, Najran Armed Forces Hospital, Ministry of Defense Health Services, Najran, SAU

**Keywords:** artificial intelligence, chatbots, chronic diseases, conversational agents, healthcare

## Abstract

Artificial intelligence (AI) is being used by an increasing number of conversational agents, sometimes known as chatbots. In applications related to health care, such as those that educate and assist patients with chronic illnesses, which are among the main causes of mortality in the 21st century, they are becoming more and more common. Chatbots powered by AI allows for more frequent and efficient engagement with these patients. This systematic review aimed to examine the traits, medical conditions, and AI architectures of conversational agents that are based on artificial intelligence and are specifically made for chronic illnesses. We searched four databases (Scopus, Web of Science, PubMed, and Cumulative Index to Nursing and Allied Health Literature [CINAHL]) to search for relevant studies using specific inclusion and exclusion criteria. Among these databases, we found 386 studies that were screened for duplicates and then assessed by inclusion and exclusion criteria. We included the 10 most relevant studies in this systemic review. There is a dearth of research on AI-based interactive agents for chronic illnesses, and what little is available is primarily quasi-experimental studies, including chatbots in prototype stages that employ natural language processing (NLP) and enable multimodal user engagement. Future studies could benefit from comparing and evaluating AI-based conversational bots within and between various chronic health disorders using evidence-based methodology. In addition to improving comparability, more structured development and standardized evaluation procedures could improve the caliber of chatbots created for certain chronic diseases and their subsequent effects on the target patients.

## Introduction and background

One of the biggest healthcare issues of the 21st century is the global increase in the prevalence of chronic diseases [1]. Chronic diseases require continuous management by both patients and medical professionals due to their persistent nature and significant impact on health-related quality of life. Conditions such as diabetes, cardiovascular diseases, and chronic respiratory disorders are particularly burdensome, often leading to functional impairment, frequent hospitalizations, and substantial medical expenses. These costs arise from ongoing medication use, specialized interventions, rehabilitative therapies, and regular monitoring, all of which are essential for disease control and complication prevention [2].

The World Health Organization (WHO) has given particular attention to digital health interventions as a way to enhance public healthcare services and achieve universal health coverage [3]. The focus of digital health interventions has shifted in recent years from traditional areas like eHealth or mobile health to the cutting edge of sophisticated computational sciences, particularly big data analytics, genomics, and artificial intelligence (AI) [4]. AI has gained a lot of traction in the healthcare industry, covering important areas including early disease identification, disease progression interpretation, treatment regimen improvement, and the development of innovative intervention techniques [5].

The use of AI-powered chatbots for digital healthcare intervention has grown in popularity due to advancements in speech recognition, natural language processing (NLP), and artificial intelligence [6]. These advanced computer algorithms are painstakingly designed to mimic and efficiently process spoken or typed human conversations. As a result, patients and healthcare professionals have the exceptional opportunity to interact with digital devices in a manner that closely resembles a conversation with a real human speaker. Recent advances in artificial intelligence have made it possible for interactions to resemble those between humans and computer agent equivalents more and more [7]. Advancements in chatbot technology have enhanced human-robot communication, enabling more sophisticated and realistic interactions. This has driven significant growth in various industries, including e-commerce, travel, tourism, and healthcare, where human-like engagement is increasingly valued [8].

AI-powered chatbots have provided significant benefits across various industries, with healthcare being a primary beneficiary [9]. They have been used to offer scalable, reasonably priced medical support services that are always available via internet platforms or mobile apps. For example, adults undergoing cancer treatment experienced lower anxiety levels when chatbots offered support and monitoring, doing away with the necessity for a medical professional's engagement [10]. By helping patients and clinicians, helping people change their behavior, and helping older persons in their homes, chatbots can significantly improve consultations. Furthermore, chatbots have the potential to be extremely important in achieving specific goals, such as self-monitoring and overcoming obstacles to self-management. In the context of managing chronic illnesses, these roles are crucial [11].

This systematic review aimed to explore the applications and challenges of AI-based chatbots in chronic disease management. By synthesizing existing evidence, this review studied existing evidence to evaluate the effectiveness, usability, and limitations of AI chatbots in chronic disease management.

## Review

Methodology

Review Protocol

This systematic review was conducted in accordance with the preferred reporting items for systematic reviews and meta-analyses (PRISMA) guidelines [12].

Search Strategy

**Figure 1:**
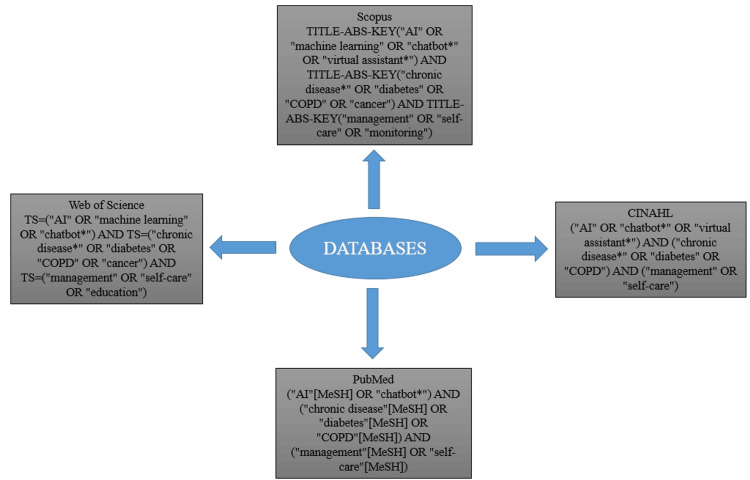
Search strategy for four different databases

The search strategy was designed to retrieve relevant studies from multiple electronic databases, including Scopus, Web of Science, PubMed, and CINAHL. The search was conducted without a specific publication time frame, allowing for a comprehensive review of the literature. A combination of Medical Subject Headings (MeSH) terms and keywords related to artificial intelligence, chatbots, and chronic diseases was used to maximize retrieval. The specific MeSH terms utilized are detailed in Figure 1.

Eligibility Criteria

The eligibility criteria were established to include studies evaluating AI-based chatbots designed specifically for chronic disease management. Eligible studies focused on chatbot applications for patient engagement, symptom monitoring, medication adherence, and lifestyle modification. Only peer-reviewed articles published in English were considered. Studies that primarily examined AI chatbots for acute conditions or general mental health support, as well as non-peer-reviewed articles, editorials, and opinion pieces, were excluded. Studies lacking quantitative or qualitative assessments of chatbot effectiveness and duplicate publications were removed.

Studies Selection

The study selection process was conducted in two stages. Initially, two independent reviewers, who were also authors of this study and had expertise in artificial intelligence and healthcare research, screened the titles and abstracts of all retrieved studies to identify those that met the inclusion criteria. Any discrepancies between reviewers were resolved through discussion or consultation with a third reviewer, who also had relevant expertise. In the second stage, full-text reviews of shortlisted articles were conducted, and studies that did not meet the eligibility criteria were excluded, with reasons for exclusion documented. The PRISMA flow diagram was used to illustrate the selection process, detailing the number of studies identified, screened, assessed for eligibility, and included in the final review.

Data Extraction

A structured data extraction process was employed to ensure consistency and completeness. A standardized data extraction sheet was used to collect key information from each included study, including study characteristics (author, year, country, study design), chatbot characteristics (AI model used, natural language processing capabilities, integration with electronic health records or wearable devices), target chronic disease (diabetes, cardiovascular disease, asthma, etc.), intervention details (mode of delivery, chatbot functionalities, level of human involvement), assessed outcomes (patient engagement, adherence, symptom control, health outcomes), and identified challenges (user experience, ethical concerns, technical barriers).

Quality Assessment

The risk of bias assessment was conducted using the risk of bias in non-randomized studies of interventions (ROBINS-I) tool. This tool evaluates bias across seven key domains: bias due to confounding, bias in the selection of participants, bias in the classification of interventions, bias due to deviations from intended interventions, bias due to missing data, bias in the measurement of outcomes, and bias in the selection of the reported result. Each study was assessed and categorized as having a low, moderate, or serious overall risk of bias. Two independent reviewers performed the assessment, and any discrepancies were resolved through discussion to ensure consistency and accuracy.

Data Synthesis

The data synthesis approach involved a qualitative narrative synthesis, categorizing studies based on chatbot applications, effectiveness, and associated challenges. While a meta-analysis was initially considered, the anticipated heterogeneity in study designs, chatbot functionalities, and outcome measures made a quantitative synthesis impractical. Therefore, findings were synthesized descriptively to provide a comprehensive overview of the applications and challenges of AI-based chatbots in chronic disease management.

Results

Search Results

**Figure 2:**
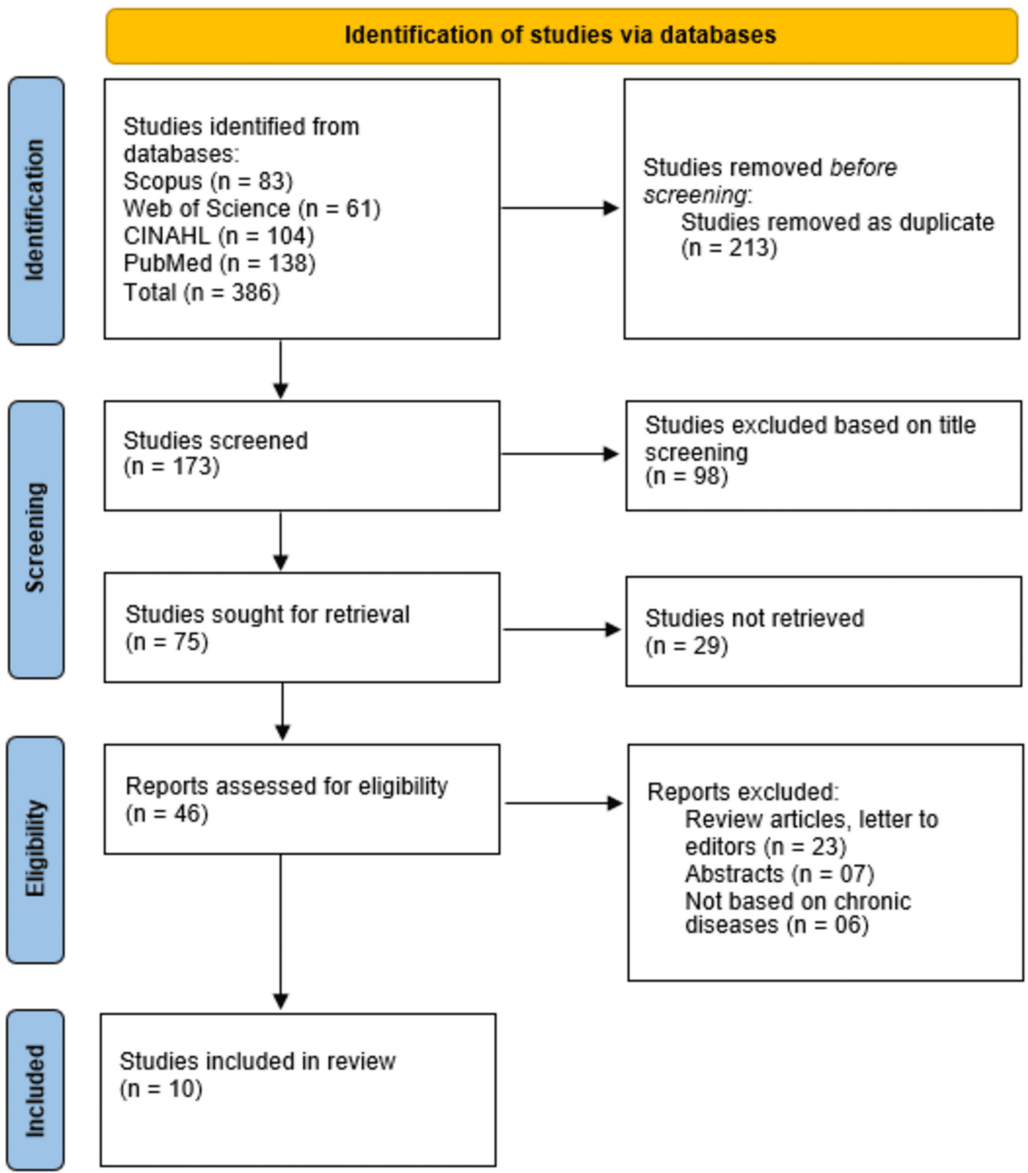
PRISMA flowchart of studies selection

A comprehensive database search identified a total of 386 studies from Scopus (n = 83), Web of Science (n = 61), CINAHL (n = 104), and PubMed (n = 138). After removing 213 duplicate studies, 173 studies remained for screening. Following title screening, 98 studies were excluded, leaving 75 studies for further retrieval. Of these, 29 studies could not be retrieved, and 46 full-text reports were assessed for eligibility. After applying the inclusion and exclusion criteria, 23 review articles and letters to the editor, seven abstracts, and six studies not focused on chronic diseases were excluded. Ultimately, 10 studies met the eligibility criteria and were included in this systematic review (Figure 2).

Characteristics of Included Studies

Table 1 shows the complete list of included studies. Eighty percent (8/10) of the articles were published after 2016, with the publication dates spanning from 2010 to 2020. There were four studies carried out in the US [17,18,21,22], two in Spain [14,20], and one each in Canada [15], Australia [19], the UK [16], and South Korea [13]. Users tested and assessed the conversational agents in the majority of quasi-experimental trials. One study was a proof-of-concept investigation, and the other two were RCTs [14,17,18].

**Table 1:** Key characteristics of included studies AI: Artificial Intelligence, AIML: Artificial Intelligence Markup Language, CBT: Cognitive Behavioral Therapy, DL: Deep Learning, ML: Machine Learning, NLP: Natural Language Processing, NLU: Natural Language Understanding, NN: Neural Networks, STT: Speech-to-Text, TTS: Text-to-Speech.

Study	Country	Study Design	Chronic Condition(s)	Sample Size	Application goal	AI technique	Name of Chatbot	Goal of Chatbot	AI system development	Key findings and outcome
Rehman et al., (2020) [13]	South Korea	Quasi-experimental study	Diabetes and glaucoma	33	Disease diagnosis	Speech recognition, ML, DL, NLP, NLU, NN	MIRA (Medical Instructed Real-Time Assistant)	Disease Diagnosis	Internal	Algorithm performance: 89% accuracy, 90% precision, 89.9% sensitivity, 94.9% specificity, and 89.9% F-measure; excellent outcomes in every area of user experience; effective disease prediction based on primary complaints
Roca et al., (2020) [14]	Spain	Proof of Concept study	Different chronic conditions, such as psoriasis	Not reported	Disease monitoring	AIML, NLP	Virtual Assistant	Disease Diagnosis	Not reported	development of a microservices-based chatbot prototype using messaging platforms
Rose-Davis et al., (2019) [15]	Canada	Quasi-experimental study	Juvenile idiopathic arthritis	06	Patient education	Not reported	JADE (Juvenile idio- pathic Arthritis Dia- logue-based Education)	Education	Internal	Using an AI-based extended argument model in a conversational agent prototype to provide patient education and positive feedback
Easton et al., (2019) [16]	United Kingdom	Quasi-experimental study	Chronic obstructive pulmonary disease	23	Self-management tool	Speech recognition	Avachat (=avatar & chat)/Ava	Support	External	Four different self-management scenarios were specified for patient assistance and positive engagement. AI-based voice recognition was found to be insufficient, thus a human wizard was used in place of it for video-based scenario testing.
Fulmer et al., (2018) [17]	United State	Randomized control trial	Anxiety and depression	74	Health assistance through many interventions, including mindfulness-based treatment and cognitive behavioral therapy	ML, Emotion algorithms, NLP	Tess	Coaching	External	Four weeks of chatbot engagement decreased anxiety symptoms, while two weeks of chatbot interaction with daily check-ins significantly decreased depressive symptoms. Over the course of more than two weeks, chatbot contact increased engagement and overall satisfaction more than control intervention
Fitzpatrick et al., (2017) [18]	United State	Randomized control trial	Anxiety and depression	70	CBT	Decision tree, NLP	Woebot	Coaching	External	Significantly lowering depression and promoting high levels of engagement, chatbot interaction is thought to be preferable to information-only control comparison.
Ireland et al., (2016) [19]	Australia	Quasi-experimental study	Parkinson and dementia	33	General dialogue with Parkinson's patients and assessment facilitation; future: patient speech and communication therapy	AIML	Harlie (Human and Robot Language Interac- tion Experiment)	education and support	External	Overall good impression, but there are technical difficulties with processing speed.
Griol and Callejas (2016) [20]	Spain	Quasi-experimental study	Alzheimer	31	Disease monitoring	STT, TTS, NLP, Speech recognition	application, conversational agent	Data collection	External	Patient feedback: good assessment, perceived potential to enhance patients' cognitive abilities; caregiver feedback: satisfactory system interaction, preference for multimodal contact because of flexibility
Rhee et al., (2014) [21]	United State	Quasi-experimental study	Asthma	15	Self-management tool	NLP	mASMAA (mobile phone-based asthma self-management aid for adolescents)	Support	Internal	Adolescent-parent partnerships are facilitated, treatment adherence and sense of control are encouraged, symptoms are the most often mentioned issue in adolescent-initiated communications, and the response rate for daily messages from teenagers is high (81%–97%).
Ferguson et al., (2010) [22]	United State	Quasi-experimental study	Heart failure	63	Self-care support	Speech recognition, NLP	Personal health management assistant	Collection of data	Internal	Development of a workable end-to-end spoken conversation system for heart failure check-up, prototype for data gathering, and adequate user involvement

Four of the 10 research projects sought to create, develop, or assess a conversational agent prototype. One study sought to create and deploy a conversational agent prototype architecture (Roca et al., 2020 [14]). One study sought to build, install, and assess a particular conversational agent (Easton et al., 2019 [16]), whereas three studies sought to solely assess one particular conversational agent (Fulmer et al., 2018 [17]; Fitzpatrick et al., 2017 [18]; Griol and Callejas, 2016 [20]). One study sought to examine a corresponding prototype and create and develop a domain-independent structure to facilitate the creation of conversational agents (Ferguson et al., 2010 [22]).

Out of the 10 studies, three failed to disclose the funding sources. No conflicts of interest were mentioned in seven studies. One study did not report on conflicts of interest (Roca et al., 2020 [14]), whereas two studies revealed pertinent conflicts of interest (Fulmer et al., 2018 [17]; Fitzpatrick et al., 2017 [18]).

Key Findings

Two investigations that evaluated the conversational agents' technical performance revealed consistently high message response rates (81-97%) and performance metrics of the conversational agents (sensitivity: 90%, accuracy: 89%, precision: 89.9%, specificity: 94.9%, and F-measure: 89.9%). User experience was evaluated in seven studies. In terms of acceptability, comprehension of the chat agents, comprehension of the responses from the systems, engagement rates, or content relevancy, the user experience was largely favorable. When compared to the control groups, two RCTs that examined health-related outcomes discovered that interacting with the conversational bots reduced symptoms of anxiety and depression.

Four studies either claimed that the conversational agent was engaging or found an elevated level of interaction with the agent. According to one study, the conversational agent prompted and encouraged treatment adherence in addition to raising knowledge of disease symptoms (Rhee et al., 2014 [21]). According to one study, telemonitoring for chronic illnesses could be provided via the built conversational agent architecture (Griol and Callejas, 2016 [20]). Health professionals also commented in the same survey that the architecture offers a versatile option for data storage and individualized monitoring services.

Risk of Bias Assessment

The ROBINS-I assessment revealed varying levels of bias among the included studies. Fulmer et al. (2018) [17] and Fitzpatrick et al. (2017) [18] demonstrated a low risk of bias across all domains, indicating well-controlled study designs. The majority of studies, including Rehman et al. (2020) [13], Rose-Davis et al. (2019) [15], Easton et al. (2019) [16], Ireland et al. (2016) [19], Griol and Callejas (2016) [20], Rhee et al. (2014) [21], and Ferguson et al. (2010) [22], exhibited a moderate overall risk of bias, with issues primarily related to confounding, missing data, or measurement of outcomes. In contrast, Roca et al. (2020) [14] had a serious risk of bias, particularly in the domains of confounding, deviations from intended interventions, and outcome measurement, which may limit the reliability of its findings. These results emphasize the need for more rigorously designed studies with improved methodological quality to strengthen the evidence base on AI-based chatbots for chronic disease management (Table 2).

**Table 2:** Risk of bias assessment using the ROBINS-I tool ROBINS-I: Risk of bias in non-randomized studies of interventions

Study	Bias due to Confounding	Bias in Selection of Participants	Bias in Classification of Interventions	Bias due to Deviations from Intended Interventions	Bias due to Missing Data	Bias in Measurement of Outcomes	Bias in Selection of the Reported Result	Overall Risk of Bias
Rehman et al., (2020) [13]	Moderate	Low	Low	Moderate	Low	Moderate	Moderate	Moderate
Roca et al., (2020) [14]	Serious	Moderate	Low	Serious	Not reported	High	High	Serious
Rose-Davis et al., (2019) [15]	Moderate	Low	Low	Moderate	Low	Moderate	Moderate	Moderate
Easton et al., (2019) [16]	Moderate	Low	Low	Moderate	Low	Moderate	Moderate	Moderate
Fulmer et al., (2018) [17]	Low	Low	Low	Low	Low	Low	Low	Low
Fitzpatrick et al., (2017) [18]	Low	Low	Low	Low	Low	Low	Low	Low
Ireland et al., (2016) [19]	Moderate	Low	Low	Moderate	Moderate	Moderate	Moderate	Moderate
Griol and Callejas (2016) [20]	Moderate	Low	Low	Moderate	Moderate	Moderate	Moderate	Moderate
Rhee et al., (2014) [21]	Moderate	Low	Low	Moderate	Low	Moderate	Moderate	Moderate
Ferguson et al., (2010) [22]	Moderate	Low	Low	Moderate	Moderate	Moderate	Moderate	Moderate

Discussion

Ten studies were found in our systematic review, the majority of which were quasi-experimental and two of which were RCTs. To the best of our knowledge, this is the only comprehensive assessment of the literature that focuses exclusively on conversational agents powered by artificial intelligence that are employed in the treatment of chronic illnesses. Other recent evaluations concentrated on conversational assistants for a particular health issue, such as mental health [23]; the broad usage of chatbots in healthcare [24]; or certain aspects of them, including technological architectures or customization [25,26].

Eighty percent of the papers we found were published in 2016 or later, highlighting the relative novelty of research in this field. Given that our search was conducted without a specific publication time frame, this finding underscores the limited number of studies available on AI-based chatbots for chronic disease management. This aligns with previous reviews that concluded conversational agents are still in their early stages but are increasingly being integrated into healthcare [24-26]. We found that the majority of AI-based conversational agents were still in the prototype stage and not yet accessible to the general public. They are employed in the diagnosis, coaching, data gathering, support, and educating of patients with long-term illnesses [16].

The underdevelopment of this field may be attributed to several factors, including the complexity of designing AI-driven chatbots that can provide reliable, personalized healthcare support, the need for rigorous validation through clinical trials, and concerns regarding data privacy and ethical considerations. Additionally, the slow adoption of emerging technologies in clinical settings and the lack of standardized frameworks for chatbot implementation may further contribute to this gap.

A growing number of conversational agents are able to provide genuine interactions between people and their artificially intelligent agent counterparts thanks to recent developments in AI software [27]. Nevertheless, issues like skewed and opaque decision-making that undermine confidence in the results are still present and have only been partially addressed. For example, AI chatbots trained on biased datasets may produce recommendations that favor certain patient demographics while neglecting others. Additionally, the lack of transparency in machine learning algorithms, often referred to as the 'black box' problem, makes it difficult for healthcare professionals to interpret and trust chatbot-generated responses. When coupled with the practical challenge of requiring huge datasets for algorithm training, this may help to explain why there are so few applications now in use [13,28].

Current chatbots use a range of communication channels, some of which are vendor-specific, such as those designed for Android smartphones [29]. We recommend that future research monitor these platform-dependent trends since they may indicate a greater reliance on technology providers or their influence over apps related to health care.

The study that was found lacked true geographical diversity, with half of the studies taking place in North America, a single one in Australia and Asia, and the remainder (30%) in Europe [30]. Not just one study was carried out in Africa. Furthermore, 90% of these study sites are deeply ingrained in Western cultures, which significantly limits how broadly the findings may be applied. Future studies should aim to encompass varied geographical areas to ensure context-specific relevance, given the global prevalence of chronic illnesses and the necessity of applying solutions relevant to healthcare systems [31]. To improve variability and generalizability, we suggest broadening the scope of research outside the Western economic-cultural environment and also incorporating growing economies like China and India.

Most of the research that was found focused on completely planning, creating, or testing a conversational agent tailored to a single chronic illness. This research implies that rather than developing universal interventions that may be used for a wide range of chronic diseases, AI-based conversational agents will eventually develop into offering specialized support for certain chronic ailments. The impact of such expertise on treatment-related metrics like patient satisfaction or adherence to treatment may be examined in future studies.

The evaluation metrics for the identified AI-based conversational bots and their impact on the specific chronic illnesses they were intended to address were wide-ranging and inconsistent. User satisfaction and chatbot engagement, two generalistic usability metrics for technical platforms, were the most often reported metrics [32]. Just two investigations evaluated the conversational agents' technical ability, and two more studies discussed the results pertaining to health. Nonetheless, the measurements and documented results were generally favorable and showed good health-related outcomes, high engagement, and a pleasant user experience in addition to excellent overall performance. To improve study quality and comparability, future research could mandate adherence to industry-standard guidelines for healthcare research, such as the transparent reporting of evaluations with nonrandomized designs (TREND) statement [33], the consolidated standards of reporting trials of electronic and mobile health apps and online telehealth (CONSORT-EHEALTH) [34], or the mobile health evidence reporting and assessment (mERA) checklist [35]. The lack of adoption of such reporting criteria at the moment may be explained by the largely quasi-experimental nature of the found literature and the ensuing inconsistency of evaluated measures.

According to our review, a wide range of chronic ailments, including those relating to the nervous system, cardiovascular system, rheumatism, autoimmune disorders, eyes, and psychology, are addressed by contemporary AI-based conversational agents [14,19,22]. This variety makes it more difficult to compare circumstances both inside and between them, even though it is instructive to possess such broad research of many illness kinds [21]. Prior to expanding their research to include comparisons between chronic diseases (e.g., respiratory vs. cardiovascular chronic conditions), future studies could focus on creating and assessing AI-based conversational agents' differences within chronic diseases (e.g., individual chatbots for asthma, chronic obstructive pulmonary disease (COPD), and sleep deprivation as manifestations of chronic respiration diseases) [14].

Adhering to such an investigation agenda may result in the creation of more reliable studies with greater expectations and findings that are more validly reported [13]. Similar issues relate to the wide range of stated health objectives; 30% of current AI-based interactive agents for long-term illnesses prioritize self-care management over helping with disease monitoring, while the remaining 70% focus on intervention objectives like general discourse, therapy, education, and diagnosis. The comparison of the current chatbots is further complicated by this discrepancy [17].

Of the papers that were examined, 20% were RCTs, 70% were quasi-experimental, and 10% were proof-of-concept. Usually cross-sectional and nonrandomized, these quasi-experimental investigations capture the initial impression of a particular moment. Future studies should focus on field tests, which are ideally planned as longitudinal studies to examine long-term effects, in order to gain a deeper knowledge of the practical impacts of AI-based conversational bots on the treatment of chronic illnesses. When thinking about the duration of chronic diseases, which usually afflict individuals for no less than 12 months but can last for a much longer time in a patient's life, this is particularly crucial [14].

It is also important to highlight that at least one author of each of the two RCTs in this evaluation disclosed a business stake in the conversational agent under investigation [15,16]. To improve the evaluation of chatbots from a purely external perspective, we would advise future studies to evaluate commercially available conversational agents without comparable corporate ties [36].

The fact that patients made up the bulk of the targeted intervention companions is not surprising, but it is a little surprising that only two conversational agents also included the patients' parents or other social contacts [15]. We wish to draw attention to the fact that patients with chronic illnesses frequently have significant effects on their immediate and larger social milieu. To better understand the social impact of chronic diseases, future interventions might take into account more human involvement. This could improve health outcomes and treatment adherence, two crucial therapy objectives [21].

Artificial intelligence techniques, including speech recognition, text-to-speech, natural language understanding, speech-to-text, and natural language generation, are all less common than natural language processing technologies [19]. While they are employed, they are far less frequent than other well-known AI methods like DL, ML, NN, and DT. The aforementioned prevalence of various interaction approaches among the reported conversational agents may help to explain this conclusion, which favors the creation and assessment of AI methods that are communication-focused [18]. Natural language production and emotion identification are currently being addressed more and more in the field of ongoing advancements in conversational bots' and humans' natural communication. It is anticipated that these developments will result in conversational agents powered by AI that can communicate with patients even more naturally than is currently feasible. Future study in this field is crucial because it could have a wide range of consequences on treatment-related effects and the interaction between patients and chatbots [16,37].

One significant risk associated with these ostensibly naturally speaking chatbots is the potential for patient harm, including serious injury or even fatal outcomes, if the chatbot provides incorrect or misleading recommendations. This concern is particularly critical when AI-generated suggestions involve important medical decisions, such as prescription combinations or dosage adjustments. Such risks raise pressing ethical and legal questions regarding accountability-who bears responsibility when an AI-driven chatbot provides harmful advice? Additionally, the absence of standardized regulatory frameworks for AI in healthcare further complicates the issue, as there is no clear consensus on oversight, liability, and the extent to which these systems should be integrated into clinical decision-making. Addressing these ethical dilemmas requires transparent AI development, rigorous validation, and mechanisms for human oversight to ensure patient safety [15]. When it comes to evaluating the technological or medical abilities of AI-based conversational agents, patients are frequently laypeople, thus, they may heed a chatbot's recommendations without seeking further medical explanation. In order to maximize patient safety, future chatbot development and related research should place a greater emphasis on eliminating such flaws and risks.

The diversity and overall superficiality of documented AI methods and systems are important factors to take into account, with the exception of the two studies creating and assessing conversational agent designs. Although the application of AI-based systems is stated clearly in all 10 investigations, the absence of technical details seriously impairs reproducibility and raises concerns over the caliber of the results that are published. This lack of information exacerbates the application barriers of AI-based systems, which include opaque and biased decision-making procedures and the ensuing distrust. Furthermore, in the context of chronic disease healthcare, it impedes the creation of a generic system architecture that may serve as an instructive framework for the creation and organization of AI-based chatbots [38]. We urge upcoming researchers to reveal all technical aspects needed to reproduce study findings and to grant access to the created AI-based conversational systems, either fully or partially. Apart from the previously mentioned standardized rules for healthcare research, future studies should utilize the established guidelines for reporting the technical aspects of AI-based conversational tools utilized in medicine and healthcare [37,39]. One could also refer to more generalized checklists like the Checklist for Artificial Intelligence in Medical Imaging (CLAIM), which evaluates the general framework of AI-related medical research and provides recommendations on what particular details about the selected AI model and its subsequent training, assessment, and performance should be reported [28]. Further, once the field has advanced and more standardized data are accessible, we advise future research to develop a foundation for chatbots powered by AI in the setting of healthcare for chronic diseases and synthesize an overall architecture.

The fact that half of the research findings in our evaluation used external systems to construct (some of) their AI design may point to a trend in the growth of open access and external software for AI-based conversational bots in healthcare. This should be taken into consideration in future studies to help clarify this strategy even further.

The taxonomy of AI-based software is unclear; although four studies explicitly identified their software as AI, there was a wide range of other categories that were also used, including natural interaction, state-of-the-art, intelligent, and totally automated. The utilization of a common word is made worse by inconsistent term usage. For the benefit of future research's comparability and clarity, we believe that the creation and application of precise terminology is beneficial.

Limitations

This review's main drawbacks include the frequency of quasi-experimental research, their heterogeneity, and the short number of included papers. This emphasizes how unique and complicated the field we searched was, which is why we didn't perform a meta-analysis. Lastly, the dependability of results in studies with a high risk of bias was diminished by the wide variations in risk of bias among the included research. This made it harder for us to believe the published results of research with a high risk of bias.

## Conclusions

The use of AI-based conversational agents in healthcare settings is growing because of technological advancements. Despite the medical predominance of chronic illnesses and their financial burden on 21st-century healthcare systems, there are currently few applications in this developing field of research that are specifically designed for them. Evidence-based assessment and comparison within and between various chronic health disorders are absent from the applications now in use that have been documented in the literature. Future studies should concentrate on following reporting and evaluation standards for technical elements like the underlying AI technology and the evaluation of the entire solution.
